# Light‐guided renal cyst fenestration during laparoscopic ureterocalicostomy in a patient with left ureteropelvic junction obstruction

**DOI:** 10.1002/iju5.12129

**Published:** 2019-11-13

**Authors:** Kazumitsu Yamasaki, Masahiro Uchida, Yukiko Nishijima, Akio Hoshi, Hiroyuki Nishiyama

**Affiliations:** ^1^ Department of Urology Tsukuba Gakuen Hospital Tsukuba Japan; ^2^ Department of Urology Faculty of Medicine University of Tsukuba Tsukuba Japan

**Keywords:** laparoscopic pyeloplasty, laparoscopic ureterocalicostomy, light‐guided laparoscopic renal cyst fenestration, renal cyst, ureteropelvic junction obstruction

## Abstract

**Introduction:**

Laparoscopic ureterocalicostomy is a useful alternative to laparoscopic pyeloplasty for treating ureteropelvic junction obstruction under certain conditions. One concern regarding this technique is the inevitability of amputation of the renal lower pole to expose the lower renal calyx.

**Case presentation:**

A 43‐year‐old man who presented ureteropelvic junction obstruction and multiple renal cysts underwent laparoscopic pyeloplasty, which could not be performed because of the intrarenal ureteropelvic junction. We switched the surgical technique to modified laparoscopic ureterocalicostomy, wherein amputation of the lower renal pole was substituted with fenestration of a renal cyst under the guidance of ureteroscopic light. Computed tomography performed 2 months postoperatively showed good patency of the anastomosis.

**Conclusion:**

Light‐guided laparoscopic renal cyst fenestration followed by ureterocalicostomy is feasible in patients with ureteropelvic junction obstruction and lower pole renal cysts adjacent to the lower renal calyx.

**Abbreviations & Acronyms**
CT: computed tomography
LFRC: laparoscopic fenestration of renal cysts
LUC: laparoscopic ureterocalicostomy
UPJ: ureteropelvic junction
UPJO: ureteropelvic junction obstruction

**Keynote message**

We report a case of a 43‐year‐old man with UPJO and multiple renal cysts of the left kidney. Laparoscopic pyeloplasty was initially planned, but the surgery was difficult because the intrarenal pelvis was completely covered by both the renal artery and vein. LFRC in the lower pole of the left kidney performed under ureteroscopic guidance followed by LUC successfully resolved the hydronephrosis. This new technique may be useful in treating some selected patients with UPJO with lower pole renal cysts, wherein performing usual laparoscopic pyeloplasty is difficult.

## Introduction

LUC is useful in treating some cases of UPJO where dismembered laparoscopic pyeloplasty is difficult to execute.^1^ While extensive excision of lower pole tissue to expose the calyceal lumen is one of the key technical facets for a successful outcome,^2^ it also might be a concern since cross‐clamping of the renal hilum is necessary to control bleeding if the renal parenchyma is not adequately thin.

## Case presentation

**Figure 1:**
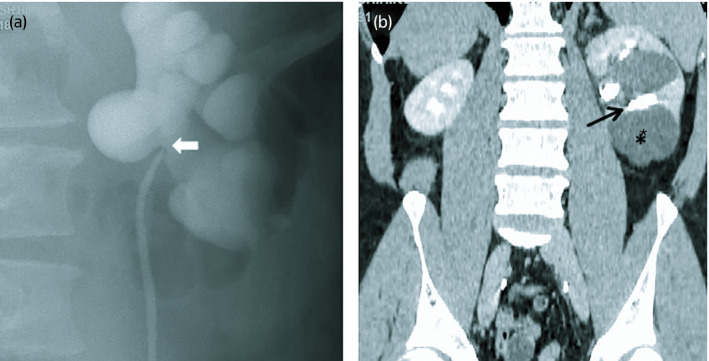
(a) Retrograde pyelogram shows left hydronephrosis with high ureteral insertion and intrarenal UPJO area (arrow). (b) Contrast‐enhanced CT‐KUB in the delayed phase. The left lower calyx (arrow) is adjacent to a renal cyst (*).

A 43‐year‐old man was presented to our hospital with left flank pain for 5 days. His serum creatinine level had increased from to 0.94 to 1.45 mg/dL obtained 15 months ago. He was suspected of having obstructive uropathy, and retrograde pyelography indicated that a left UPJO existed and that the site of obstruction might be intrarenal (Fig. 1a). The patient subsequently underwent insertion of a double‐J ureteral stent, which resulted in recovery from uropathy. Contrast‐enhanced CT showed dissolution of the left hydronephrosis and large renal cyst(s) directly adjacent to the left lower renal calyx (Fig. 1b). Six weeks after removal of the double‐J stent, he underwent surgery for the left UPJO.

## Procedure

**Figure 2:**
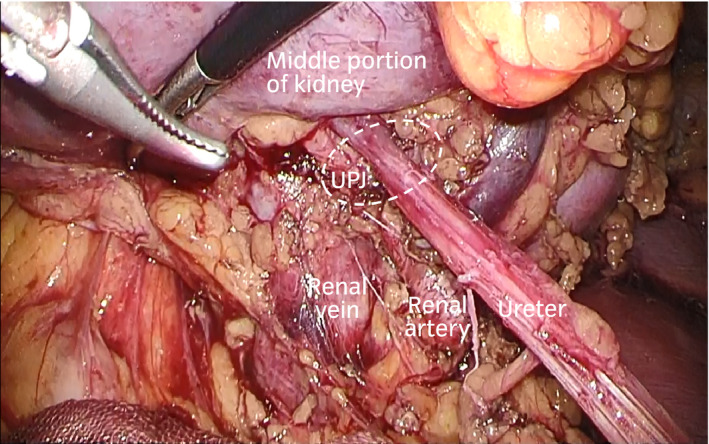
Intraoperative image showing intrarenal UPJ adjacent to the renal artery and vein.

**Figure 3:**
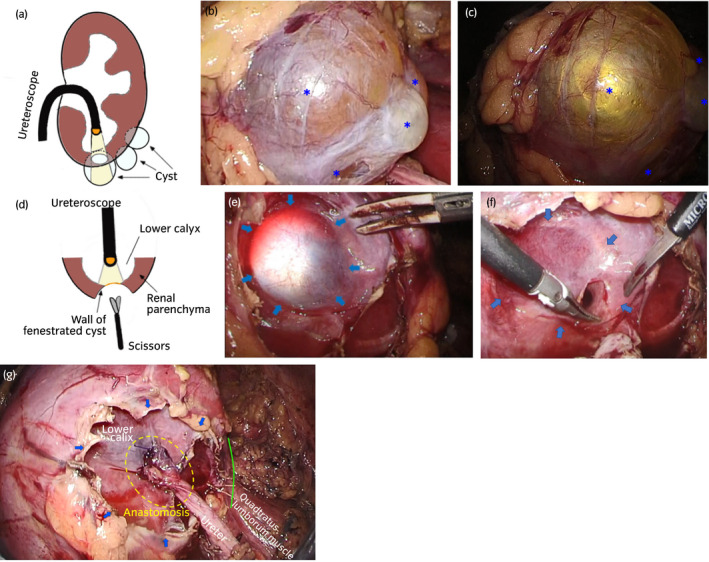
(a) Illustration showing how to get direct access to the lower calyx by LFRC, and (b) an intraoperative image of lower pole renal cysts (*) observed under usual laparoscopy (c) and with a help of illumination by the ureteroscope. (d) Illustration showing how the lower calyx looked like after LFRC. (e) An intraoperative image of the outer surface of the lower renal calyx after LFRC illuminated by the ureteroscope. Arrows indicates the border between the renal parenchyma and lower renal calyx. (f) Outer surface of the lower renal calyx right after fenestration of the calyceal wall. Note that the opening was located close to the border between the renal parenchyma (arrow). (g) End of the surgery. Green curve indicates the suture between the cyst wall and quadratus lumborum muscle. Arrows indicate the edge of the cyst wall.

After general anesthesia, he was placed in a lateral flank position. He underwent transperitoneal laparoscopic pyeloplasty using a four‐port technique, as described previously.^3^ Upon dissection of the upper ureter close to the renal hilum, the UPJ was revealed to be intrarenal and adjacent to the renal artery and vein, which forced us to switch the surgical technique to LUC (Fig. 2). We considered obtaining direct access to the calyx by fenestrating the renal cyst adjacent to the calyx instead of renal lower pole amputation. To ensure that this idea was feasible, we used a flexible ureteroscope (Fig. 3a). The ureteroscope was transurethrally inserted into the left lower renal calyx, and a laparoscopic camera detected the strong light of the ureteroscope through the wall of the lower pole renal cyst, indicating direct contact of the lower calyx with the cyst (Fig. 3b,c). After fenestration of the renal cyst, the wall of the lower renal calyx was exposed, and the border between the calyx and renal parenchyma was illuminated by the light (Fig. 3d,e). The outer wall of the dilated lower calyx was excised to create an opening close to the renal parenchyma to receive a good blood supply (Fig. 3f). The ureter was spatulated and then transected. Anastomosis of the calyx and ureter was performed using two running sutures, and a double‐J ureteral stent was positioned in an antegrade manner via one of the trocars before starting the second suture. The wall of the fenestrated renal cyst was hitched caudally to the fascia of the lumbar quadrate muscle to assure tension‐free anastomosis (Fig. 3g).

## Outcome and follow‐up

**Figure 4:**
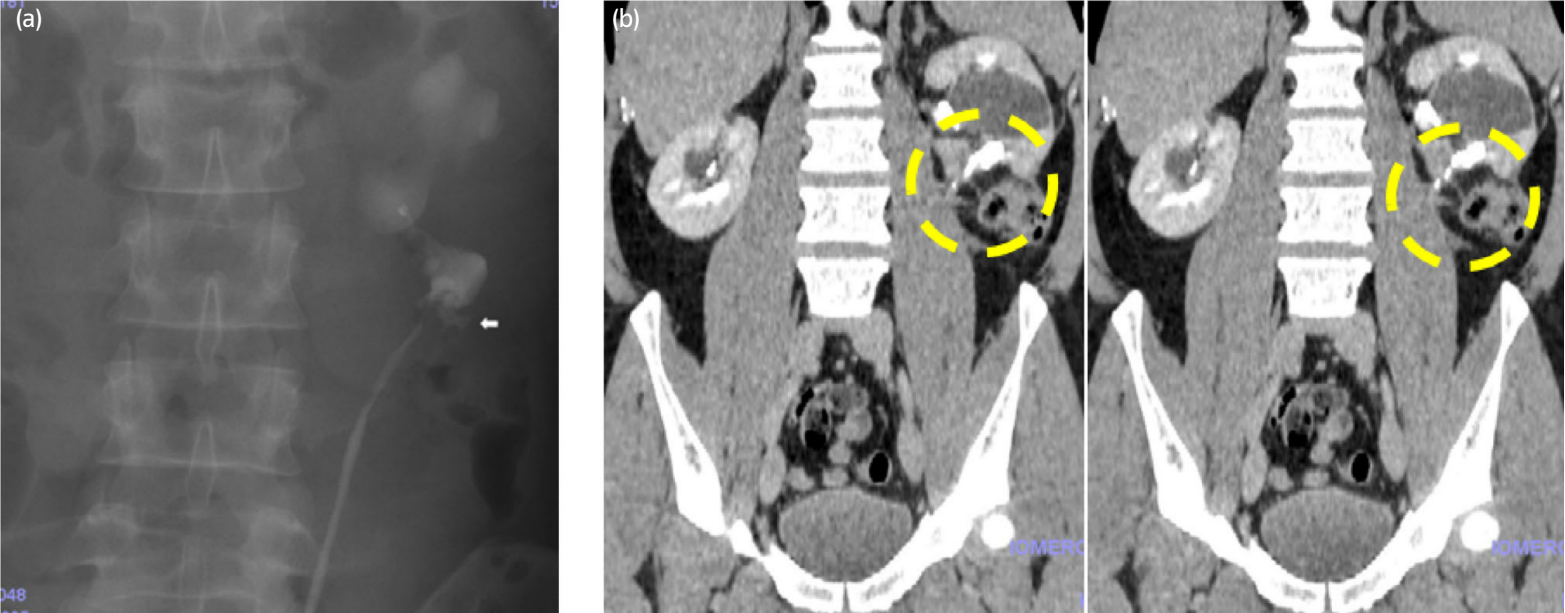
(a) Postoperative retrograde pyelogram at 1 month shows free flow of contrast medium into the renal collecting system and a leak from the site of anastomosis (arrow). (b) Postoperative contrast‐enhanced CT (CT‐KUB) at 2 months shows patency of the anastomosis (dotted circle) with no leakage.

Total operative time was 5 h and 4 min, and blood loss was estimated to be 100 ml. The Foley catheter was removed on postoperative day 9 when the total amount of ascites was almost zero, and the information drainage tube was removed on day 11. A retrograde ureteropyelogram at 4 weeks confirmed patency of the anastomosis and a tiny collection of contrast medium around the anastomosis site, which we did not recognize as a leakage, but a part of the lower calyx previously was hidden by the retraction caused by a huge renal cyst (Fig. 4a). A 2‐month postoperative contrast‐enhanced CT scan showed complete disappearance of the leakage and patency of the anastomosis (Fig. 4b). His postoperative serum creatinine level remained in the normal range.

## Discussion

Anderson–Hynes dismembered pyeloplasty has been the most common open and laparoscopic surgical procedure for UPJO repair with long‐term cure rates of >90%.^4^ Dismembered pyeloplasty may not be the best choice, however, in some complicated cases such as those with recurrent UPJO and the scarred or intrarenal pelvis.^5^ In such specific cases, LUC may be performed with good outcomes.^6^ When performing the procedure, however, it must be noted that lower pole amputation might result in hilar occlusion and sacrifice of normal renal parenchyma.^6^ In very restricted cases similar to ours, in which extrarenally protruding lower pole renal cyst(s) are located adjacent to the lower pelvic calyx, LFRC followed by LUC can be a good strategy to circumvent the concerns mentioned above. To the best of our knowledge, this is the first report describing this technique for UPJO treatment.

Although LFRC itself is a straightforward procedure, the renal cyst(s) most suitable for fenestration should be carefully selected for successful anastomosis. As shown in this case, light‐guidance using a ureteroscope may be a good method for this purpose. If performing LUC following LFRC is anticipated, we recommend inserting a ureteroscope in the supine or lithotomy position before starting surgery, since its insertion in a lateral flank position might render the operation more difficult to perform.

Another concern in performing LUC after LFRC is the fragility of the anastomosis. In conventional LUC, an anastomosis is achieved by suturing the ureter and renal calyx with renal parenchyma in the whole circumference. In this new technique, however, the ureter is anastomosed with the renal calyx only partially combined with the renal parenchyma. This fragility may cause a delay in wound healing at the anastomosis site, which may be a reason for prolonged urine leakage observed even 4 weeks postoperatively. We speculate that tension‐free anastomosis achieved by hitching the lower pole is very important in adding to the stability of the anastomosis; however, we recommend a longer indwelling time of the double‐J stent compared with that for conventional LUC.

## Conclusion

To the best of our knowledge, this is the first report of successful execution of LFRC followed by LUC for UPJO. This new technique is useful and straightforward, although its indication is restricted. For best results with this technique, the renal cyst to be fenestrated should be carefully selected by ureteroscopic observation in order to achieve tension‐free anastomosis during the procedure.

## Consent of the patient

Consent was obtained from the participant.

## Conflict of interest

The authors declare no conflict of interest.
